# Unexpected Relapse: Insights Into Granulomatosis With Polyangiitis

**DOI:** 10.7759/cureus.56883

**Published:** 2024-03-25

**Authors:** Zeyad J Rifai, Akshay Kohli, Samie Gilani, Xueguang Chen

**Affiliations:** 1 Department of Internal Medicine, Southern Illinois University School of Medicine, Springfield, USA; 2 Department of Internal Medicine, Division of Pulmonology and Critical Care Medicine, Southern Illinois University School of Medicine, Springfield, USA; 3 Department of Internal Medicine, Division of Nephrology, Southern Illinois University School of Medicine, Springfield, USA

**Keywords:** c-anca/proteinase 3 (pr3)-positive granulomatosis with polyangiitis (gpa) (formerly known as wegener's granulomatosis), acute kidney injury, relapsing illness, renal replacement therapy (rrt), complement inhibitor, uremic patients, peripheral pulmonary nodule, anca-associated vasculitis

## Abstract

Granulomatosis with polyangiitis (GPA) is a rare vasculitis that can pose a significant mortality risk given its multiorgan involvement and is the most common of the three anti-neutrophil cytoplasmic antibodies (ANCA)-associated vasculitides. Cardinal pathological features include necrotizing granulomas of the respiratory tract, small and medium vessel vasculitis, and glomerulonephritis. Early treatment is imperative to reduce permanent organ damage such as end-stage kidney disease. We describe the first case of GPA relapse 38 years after the initial pulmonary presentation. The patient previously had isolated lung involvement with preserved renal function, but presented with an acute kidney injury, uremia, and several constitutional symptoms. The patient was treated with corticosteroids and intermittent hemodialysis and initiated on immunosuppressants; the clinical course is highlighted by eventual renal recovery. Our purpose is to highlight the importance of treating patients to complete immunological recovery, particularly in GPA vasculitis, to prevent unnecessary relapse and further loss of renal function.

## Introduction

Granulomatosis with polyangiitis (GPA) is a small and medium vessel vasculitis with multiorgan involvement associated with the presence of anti-neutrophil cytoplasmic antibodies (ANCA) [1-3]. The survival rate for GPA has improved with recent usage of immunosuppressants and biologics; however, an increased risk of relapse has been associated with cavitary lung lesions, seropositivity, and increasing ANCA titers, among other factors [1-3]. The incidence of GPA relapse is estimated to be roughly 13% within 36 months [3]. Many utilize the French vasculitis relapse score at the time of diagnosis to assess the relapse risk; however, the prevalence of relapse varies widely in the documented literature [1-6]. This is important as the survival rate of relapse-free patients over a nine-year span is 25%, while patients who experience relapse with severe renal involvement have a significantly elevated rate of mortality [7]. Overall, the incidence of GPA vasculitis worldwide is estimated to be 10 individuals per one million cases [7].

The hallmark organs involved in GPA vasculitis are the upper respiratory tract, lungs, and kidneys [7]. The presentation of GPA typically involves the upper respiratory tract, with 90% of patients complaining of nasal ulcerations, nasal and sinus pain, purulent discharge, and epistaxis, among other conditions [7]. Multiple case reports also mention ocular and otic involvement as the presenting symptoms of GPA [6]. Pulmonary parenchymal or airway involvement varies significantly from hoarseness to alveolar hemorrhage [8]. Alveolar hemorrhage is a life-threatening manifestation that is estimated to occur in 5-45% of patients with ANCA vasculitis, commonly presenting as dyspnea and hypoxemia [8,9]. Renal involvement as an early presentation is noted in only 20% of patients. However, 80% of patients have renal involvement within two years of symptom onset [7]. The most common renal presentation is progressive crescentic glomerulonephritis, resulting in late-stage chronic kidney disease or end-stage renal disease [7]. We present the first case of GPA relapse wherein our patient presented more than three decades following the initial presentation of lower respiratory tract involvement.

## Case presentation

**Figure 1:**
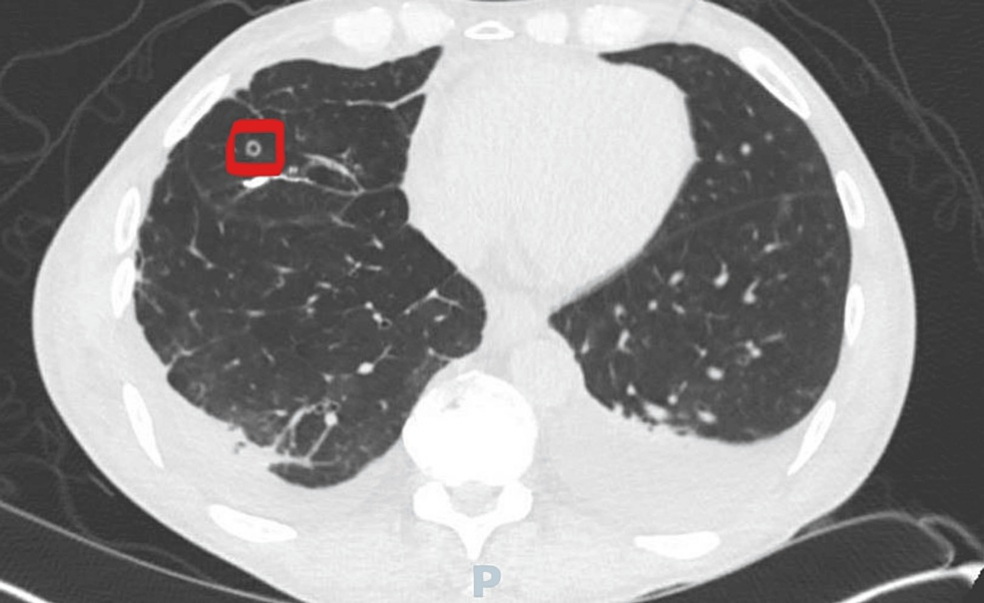
Cavitary lesion (6 mm) of the anterior right lung, consistent with quiescent granulomatosis with polyangiitis (GPA)

A 69-year-old male, with a documented medical history of GPA confirmed through a lung biopsy conducted in 1985, is included in this report. Furthermore, it is noted that the patient has maintained consistent renal function. He presented to his primary care physician's office with a 10-day complaint of weight loss, frank hematuria, diarrhea, and increased urinary frequency. He was advised to present to the emergency department after routine lab work revealed a serum creatinine of 8.2 mg/dL (baseline 0.8 mg/dL). Urinalysis revealed 106 red blood cells/high powered field, large hemoglobinuria, and without proteinuria. Computed tomography (CT) of the chest obtained in the emergency department revealed a cavitary lung nodule, measuring 6 mm in diameter, of the anterior right lung (Figure 1). A pulmonologist was consulted to evaluate the lung lesion and determined it to be a quiescent state lesion consistent with the patient’s history of GPA, without evidence of pulmonary hemorrhage. Autoimmune and paraproteinemia workup revealed a cytoplasmic ANCA (C-ANCA) titer of 1:640, as well as positive antibodies against proteinase-3 (PR3). The patient’s uremic milieu of singultus, weight loss, fatigue, and nausea prompted the initiation of intermittent hemodialysis.

**Figure 2:**
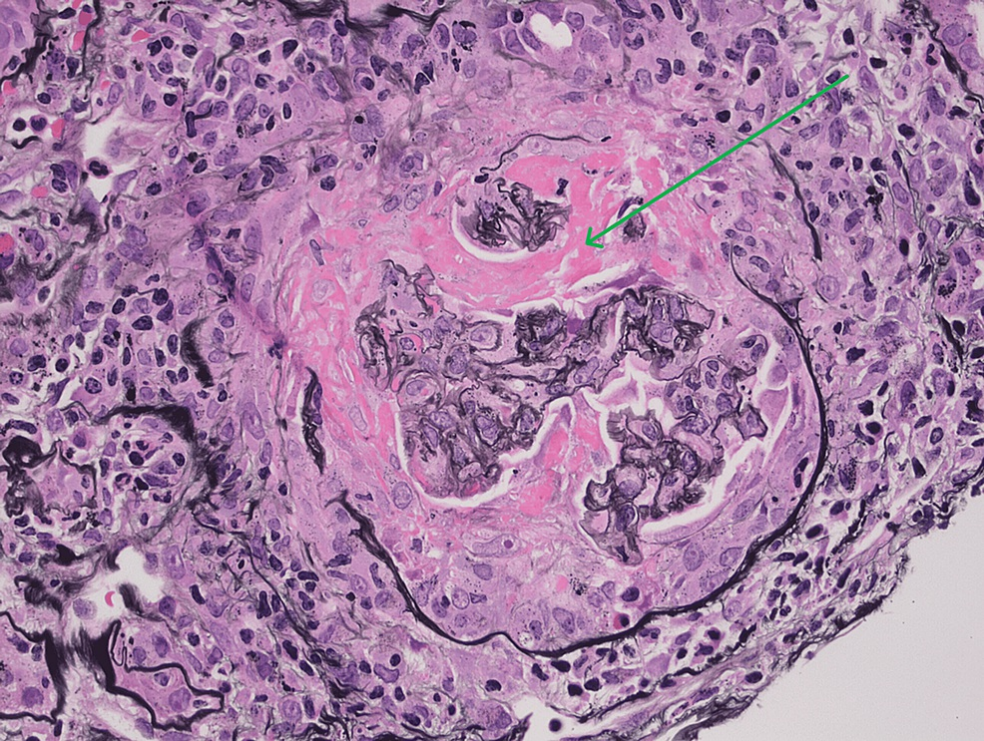
Silver stain, pauci-immune necrotizing and crescentic glomerulonephritis, with fibrinoid necrosis and collapse of the glomerular tuft

**Figure 3:**
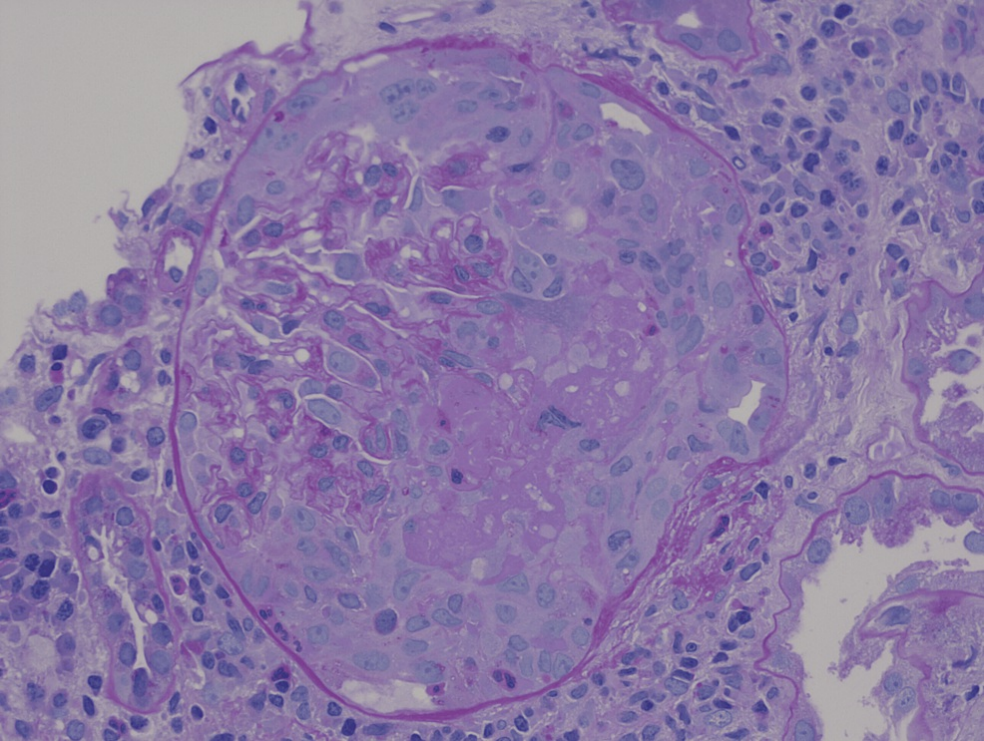
High power, periodic acid Schiff’s (PAS) stain, pauci-immune necrotizing and crescentic glomerulonephritis, with fibrinoid necrosis and collapse of the glomerular tuft

**Figure 4:**
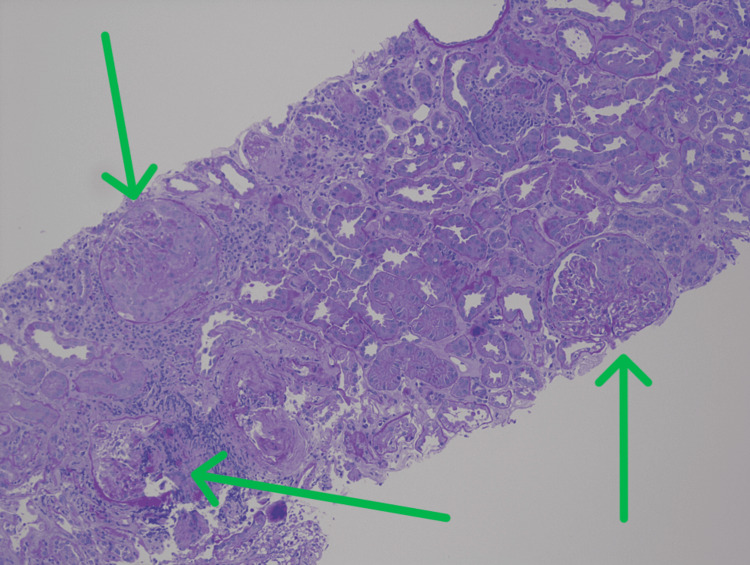
Low power, PAS stain, pauci-immune necrotizing and crescentic glomerulonephritis, with fibrinoid necrosis and collapse of the glomerular tuft

A renal biopsy was consistent with diffuse necrotizing and crescentic glomerulonephritis, pauci-immune rapidly progressive glomerulonephritis (RPGN) secondary to GPA relapse (Figures 2-4). He was started on a moderate dose of methylprednisolone, underwent induction with a cyclophosphamide regimen, and received a prophylactic trimethoprim-sulfamethoxazole regimen against *Pneumocystis jirovecii* pneumonia. Following the initiation of immunosuppression and intermittent hemodialysis, there was a notable clinical improvement. The patient was discharged home with a prescription for intermittent hemodialysis, prophylactic antibiotics, and a prednisone regimen.

Outpatient follow-up was notable for renal recovery with serum creatinine trending towards baseline, as well as discontinuation of hemodialysis six weeks after initial renal injury. The most recent set of labs indicated chronic kidney disease stage G3bA1 with a new baseline serum creatinine of 1.6-1.9 mg/dL. Our patient was initiated on rituximab for maintenance therapy and has scheduled follow-ups with nephrology and rheumatology clinics to monitor for hematuria and then ANCA titers every six months, with the goal of treating the patient to absolute immunological recovery.

## Discussion

Relapse of GPA vasculitis is defined as evidence of glomerular hematuria that may be accompanied by worsening serum creatinine, sinopulmonary involvement, or necrotizing vasculitis on biopsy of any tissue [5]. Literature suggests multiple risk factors for GPA relapse, including seropositivity for PR3-ANCA, persisting PR3-ANCA titers, and involvement in the upper respiratory tract prior to remission [5]. Rates of relapse in GPA vasculitis vary widely in the literature attributable to selection in maintenance therapy [9-12]. In the ANCA-associated vasculitis (RAVE) trial, the rate of relapse within 18 months was 30% following cessation of rituximab [13]. There are very few documented cases of such biopsy-confirmed, delayed GPA relapse, and there is no literature regarding rates of relapse decades following initial diagnosis. Our case is the first case of GPA relapse more than three decades after initial diagnosis. There is a notable case report of GPA relapse 13 years after initial presentation; however, this case was documented in a post-renal transplant patient [14]. There is an additional case report of GPA relapse in a patient 20 years after the initial presentation. Unfortunately, the patient expired from infectious complications [15]. We describe the first case of GPA relapse 38 years after the initial presentation, highlighted by renal recovery and prefaced with the goal of serological monitoring.

Multiple trials guided our patients' care plan, some of which are to be discussed below. These trials, in addition to our central theme of the pursuit of absolute immunological recovery, aimed not only at clinical remission, but also the resolution of immune dysregulation causing the underlying disease is the central purpose of our discussion. This is of particular importance for our patient as PR3-ANCA patients are nearly twice as likely to relapse as myeloperoxidase ANCA-positive patients [3,5]. We would like to share the rationale for immunosuppressive maintenance selection and subsequent surveillance with the goal of immunological resolution.

The RAVE trial suggests that rituximab was more efficacious than a cyclophosphamide-based regimen with regard to disease relapse-remission while effectively reducing the rate of significant renal disease and alveolar hemorrhage. A key component of this trial, with regard to our patient and discussion, was the assessment of PR3-ANCA response in the rituximab arm when compared to cyclophosphamide. Specifically, 50% of patients in the rituximab arm were negative for PR3-ANCA compared to 17% in the control group at six months, with a statistically significant p-value [13]. Ideally, the patient’s ANCA titers would be monitored for 18 months to confirm absolute immunological recovery, as opposed to the six months of monitoring conducted in this trial.

Deferment of plasma exchange for our patient’s confirmed GPA vasculitis was based on the results of the plasma exchange and glucocorticoid for treatment of ANCA-associated vasculitis (PEXIVAS) trial [16]. There is no evidence-based role for plasmapheresis in this clinical context, given the lack of pulmonary hemorrhage, and we argue that the pursuit of plasma exchange delays the proper, aggressive inhibition of CD20-induced, complement-mediated cytotoxicity. Repeat ANCA titers are essential in guiding management as persistent ANCA titers, despite maintenance therapy, should prompt initiation of other anti-CD20 monoclonal antibodies, such as obinutuzumab or ofatumumab [17].

The role of high-dose corticosteroids in ANCA-mediated vasculitis has been a topic of conversation given the associated risk of infection, osteoporosis, increase in blood pressure, and other complications with corticosteroid use. The “avacopan for the treatment of ANCA vasculitis” is an encouraging trial that highlights the trend towards individualized medicine in our ever-evolving landscape. This trial suggests the noninferiority of adjuvant avacopan in the treatment of ANCA vasculitis when compared to an adjuvant corticosteroid regimen [18]. Avacopan therapy was considered for our patient in combination with his rituximab regimen. However, the cost of the new drug, when compared to readily available corticosteroids, ultimately directed our management.

The choice of maintenance therapy may be determined by the patient's tolerance to a particular regimen. Multiple studies have compared cyclophosphamide, azathioprine, methotrexate, mycophenolate, and rituximab as maintenance therapies, with the general consensus of two years of maintenance therapy [8-11]. However, despite clinical improvement, it is imperative that absolute immunological resolution is achieved [19]. Periodic monitoring of ANCA titers should be considered in all patients with a diagnosis of GPA. An increase in ANCA titers should not be the sole basis for GPA relapse treatment, and the diagnostic criteria previously mentioned must be considered [1]. Notably, Tomasson et al.'s trial illustrated that a rise in an ANCA titer had a relapse specificity of 82% [2]. This discussion is limited as this is a single case of GPA vasculitis relapse, 38 years after presentation; however, this is the first case described with this unusually delayed presentation. Our patient did not have the benefit of B-cell depletion in the year 1985, as these previously mentioned regimens were not available at the time.

## Conclusions

The diagnosis of GPA mandates lifelong monitoring due to its elevated risk of relapse and mortality, irrespective of renal involvement. Our patient serves as a poignant illustration of this necessity, being the first documented instance of GPA relapse after more than three decades of quiescence. Consistent surveillance of ANCA titers has paramount significance, aiding clinicians in swiftly detecting relapse, especially crucial in PR3-ANCA-positive patients due to their heightened relapse susceptibility. While the optimal interval for periodic serologic testing remains inadequately explored, further research is warranted to refine patient care and enhance outcomes. Notably, the rate of relapse remains high under the standard two-year treatment regimen. We advocate for a paradigm shift towards treating patients to complete immunological recovery, exemplified by sustained negative ANCA titers for 18 months, as the standard approach for both initial treatment and relapse cases. This proactive strategy aims to mitigate disease recurrence and improve long-term prognosis, emphasizing the importance of individualized management guided by objective markers of remission. Furthermore, the successful outcome observed in our patient, marked by renal recovery and discontinuation of renal replacement therapy, underscores the importance of early intervention and tailored immunosuppressive therapy. The integration of novel therapeutic modalities, such as rituximab, and the consideration of emerging treatments, such as avacopan, exemplify the evolving landscape of GPA management.
